# Bidirectional associations between sleep quality/duration and multimorbidity in middle-aged and older people Chinese adults: a longitudinal study

**DOI:** 10.1186/s12889-024-17954-8

**Published:** 2024-03-05

**Authors:** Xiaoran Wang, Rui Wang, Dan Zhang

**Affiliations:** 1 Institute of Hospital Management/Shenzhen International Graduate School, Tsinghua University, Shenzhen, China; 2 School of Nursing, Fudan University, Shanghai, China

**Keywords:** Multimorbidity, Sleep quality, Sleep duration, Bidirectional association, CHARLS

## Abstract

**Background:**

Multimorbidity and sleep disorder possess high incidence rates in the middle-aged and older people populations, posing a significant threat to quality of life and physical and mental health. However, investigators have previously only analysed the unidirectional association between sleep status and multimorbidity. We aimed to investigate bidirectional associations between sleep quality or duration and multimorbidity in middle-aged and older Chinese adults from a longitudinal perspective.

**Method:**

We enrolled a total of 9823 participants 45 years and older from the China Health and Retirement Longitudinal Study from 2015 to 2018 in our study. Multimorbidity was defined as two or more coexisting chronic diseases in the same individual based on 14 self-reported disease questions. Sleep quality was classified as “good” (restless < 1 day per week) and “poor” (restless ≥ 1 days per week); and sleep duration was divided into short (< 6 h), medium (6–9 h), and long (> 9 h). The bidirectional association between multimorbidity and sleep condition was examined using multivariate logistic regression models with adjustments for covariates.

**Results:**

Individuals with poor sleep quality showed a significantly higher prevalence of multimorbidity in the future. The adjusted OR (95% CI) values of individuals with poor sleep quality with respect to developing two diseases, three diseases, and ≥ 4 diseases were 1.39 (1.19, 1.63), 1.56 (1.23, 2.03), and 2.36 (1.68, 3.33), respectively. In addition, individuals with multimorbidity exhibited a significantly higher risk of poor sleep quality in the future. Short sleep duration led to multimorbidity in the future (OR = 1.49; 95 CI%, 1.37–1.63), while multimorbidity contributed to short sleep duration (< 6 h) in the future (OR = 1.39; 95% CI, 1.27–1.51) after full adjustment.

**Conclusions:**

There was a bidirectional association between sleep quality or short sleep duration and multimorbidity in middle-aged and older Chinese adults. We recommend that greater attention be given to clinical management among adults with sleep disorders or physical multimorbidities.

**Supplementary Information:**

The online version contains supplementary material available at 10.1186/s12889-024-17954-8.

## Introduction

Due to the continuous ageing of the population and improvements in the diagnosis and treatment of diseases, multimorbidity has become a major public health concern in recent years. Multimorbidity is defined as two or more coexisting chronic diseases in the same individual [1], and it is common among middle-aged and older Chinese adults. The prevalence of multimorbidity was reported to be 20.5% among middle-aged adults in China and 47.5% among older people [2], and there is considerable evidence that multimorbidity leads to various adverse outcomes such as a poor quality of life, high risk of death, and a large economic burden [3–5]. Therefore, it is necessary to identify risk factors for multimorbidity to generate better prevention strategies.

The problem of sleep disorder is very common in middle-aged and older adults [6]. Evidence has indicated that 37.4% of middle-aged and older people in China have possess sleep quality [7] and that sufficient sleep duration and good sleep quality are associated with favourable human health and overall well-being [8].

There is a correlation between sleep health and multimorbidity. On one hand, sleep-quality issues or short sleep duration may cause some cellular processes such as degenerative intracellular processes, oxidative stress, autonomic activity, dysregulated appetite-regulating hormones, inflammation, allergic and epigenetic processes, which are associated with multimorbidity. On the other hand, one of the factors increasing risk of disturbed sleep in older adults is consequence of multimorbidity, such as medication use, psychosocial factors [9]. Previous authors have demonstrated that sleep quality and duration are associated with specific chronic diseases or multimorbidity [10, 11]. For example, sleep-quality issues are more prominent in people with COPD [12], chronic kidney disease [13], and hypertension [14], and sleep quality and sleep duration are associated with an elevated risk of some common diseases [15, 16]. Although these findings suggest a bidirectional association between sleep condition and multimorbidity, most current research has focused on the unidirectional relationship between sleep quality or duration and specific chronic diseases or multimorbidity, and to our knowledge, there is no extant study that explores the bidirectional association between these elements.

Clarifying the relationship between sleep health and multimorbidity is important given that an increasing proportion of the global population is aging and multimorbidity heavily increases the economic burden on families and society. Sleep health may represent a modifiable risk factor potentially to prevent or reduce a number of diseases through intervention. Multimorbidity can be used as an influence on sleep health, so more sleep interventions will be given to patients with multimorbidity to improve their quality of life.

We therefore in the present study analysed data from the China Health and Retirement Longitudinal Study (CHARLS) to explore a bidirectional relationship between sleep quality/duration and multimorbidity.

## Methods

### Study population and data selection

This investigation was conducted using the longitudinal harmonised data from the China Health and Retirement Longitudinal Study (CHARLS), a nationally representative longitudinal survey of Chinese aged 45 years and older and their spouses; it includes assessments of economic standing, physical and psychological health, demographics, and social networks. More details of the CHARLS survey have been described elsewhere [17].

**Fig. 1:**
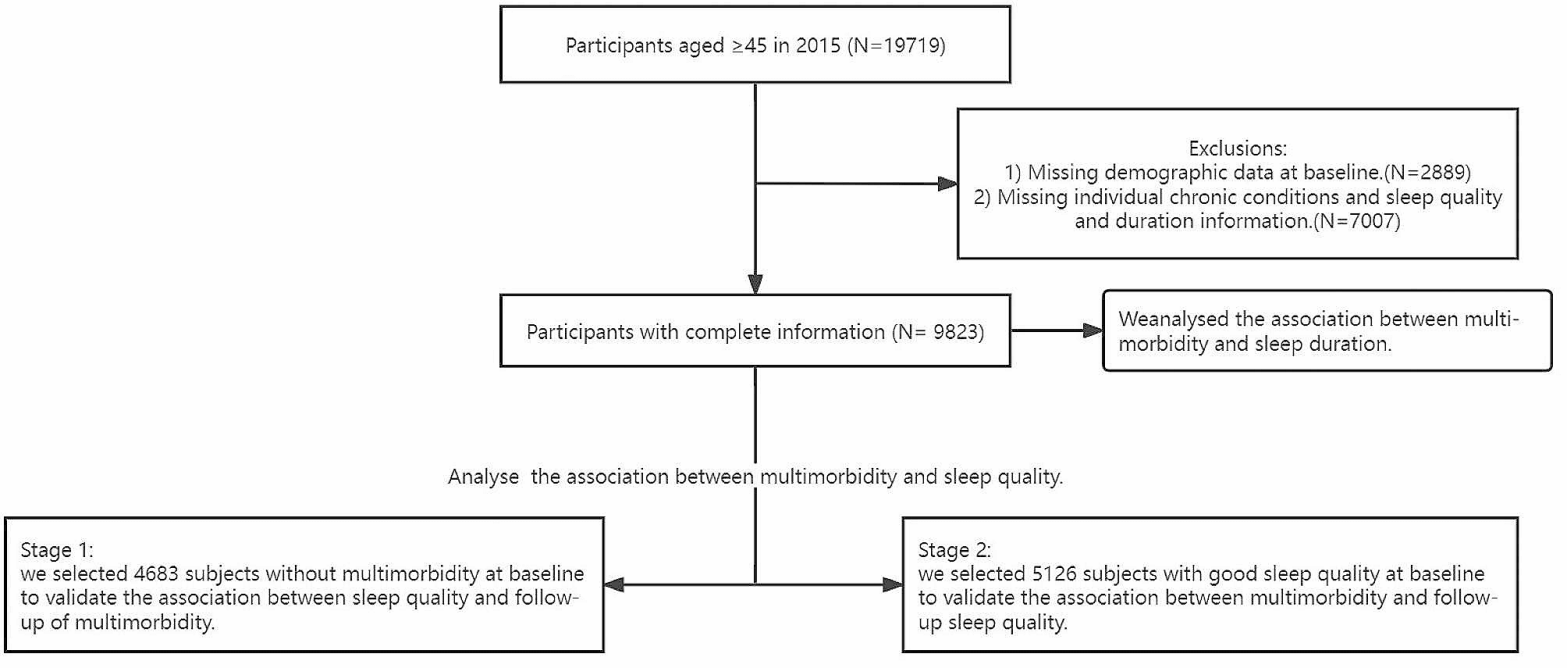
Flowchart of the selection of participants

Individuals contributing to the CHARLS data from 2015 to 2018 were enrolled in our study. We enlisted a total of 19,719 participants aged ≥ 45 years in 2015 as baseline, and these participants returned for at least a second examination in 2018. To better validate the bidirectional association between sleep condition and multimorbidity, we employed the following inclusion criteria: individuals (1) with complete important demographic data such as sex, educational status, and region of residence and (2) who answered the question on chronic condition, sleep quality, and duration. Exclusion of participants with missing demographic data and key information, a total of 9823 participants were ultimately enrolled for the 2015 baseline survey to examine the relationship between sleep health and multimorbidity. In stage 1, subjects without multimorbidity at baseline were selected to validate the association between sleep quality and follow-up of multimorbidity, enrolling a total of 4683 individuals. In stage 2, we selected 5126 subjects with good sleep quality at baseline to validate the association between multimorbidity and follow-up sleep quality. As we found it difficult to select the baseline data with respect to sleep duration, we used whole baseline data and follow-up sleep duration/multimorbidity conditions to analyse associations and adjusted for baseline chronic conditions/sleep duration to ensure the accuracy of the results (a detailed flow chart of the data selection is shown in Fig. 1). The CHARLS study was approved by our Ethics Review Committee, and informed consent was signed by all participants.

### Assessment of multimorbidity

We identified the multimorbidity of subjects according to 14 self-reported disease questions that included “Had you been diagnosed with the following conditions by a doctor: hypertension, dyslipidemia, diabetes or high blood sugar, cancer or malignant tumor, chronic lung disease, liver disease, heart problems, stroke, kidney disease, stomach or other digestive diseases, arthritis or rheumatism, or asthma?” Multimorbidity was defined as two or more coexisting chronic diseases in the same individual.

### Assessment of sleep quality and duration

Sleep quality and duration were determined by the comments/question: “My sleep was restless in the last week.” and “During the past month, how many hours of actual sleep did you get at night?” The response options regarding self-reported sleep quality included rarely or none of the time (< 1 day), some of the time (1–2 days), occasionally or a moderate amount of the time (3–4 days), and most or all of the time (5–7 days). We classified sleep quality into “good” (rarely or none of the time, < 1 day per week) and “poor” (≥ 1 day per week) [18]. Based on previous studies [19, 20], night-time sleep duration was divided into short (< 6 h), medium (6–9 h), and long (> 9 h). Hereafter, our discussion of sleep quality and duration refers to self-reported measures.

### Covariates

The covariates in this study were divided into socio-demographic, health behaviour, and lifestyle variables. Socio-demographic variables encompassed age, sex, educational level, region of residence, marital status, and type of residential address. Health behaviour characteristics included body mass index (BMI), smoking status, and alcohol consumption, and lifestyle characteristics entailed social interactions. Age in years was categorised into 45–64- and ≥ 65-age groups. We divided educational level into no completion of primary school, sishu/home school/elementary school, middle school, and high school and above, while marital status was classified as married (married or partnered) and other (separated, divorced, widowed, or never married). Type of residential address denoted family housing or another type, and nursing home or hospital. BMI was classified as underweight (less than 18.5 kg/m^2^), normal weight (18.5–24.9 kg/m^2^), overweight (25-29.9 kg/m^2^), and obese (equal to or over 30 kg/m^2^). Smoking status was categorised as non-smoker and current or former smoker, alcohol consumption as non-drinker and current or former drinker, and social participation as never (none within the previous month) or at least once per month. We measured all covariates at baseline in 2015.

### Statistical analysis

Categorical variables were reported as frequencies and percentages, and baseline characteristics were compared using Chi-squared tests. For sleep quality, we adopted binary logistic regression models in stage 1 to analyse the association between baseline sleep quality and follow-up multimorbidity. To distinguish the effects of sleep quality on a number of chronic diseases, we conducted multi-nomial logistic regression models for participants with different numbers of chronic diseases noted at follow-up (0 or 1, 2, 3 and ≥ 4). In stage 2, we constructed binary logistic regression models to examine whether baseline multimorbidity led to poor sleep quality, and we similarly executed independent analyses for participants with different number of diseases (0 or 1, 2, 3 and ≥ 4) at baseline. For sleep duration, multi-nomial logistic regression models were exploited to estimate the association between sleep duration and multimorbidity. We presented our results in the form of odds ratios (ORs) with 95% confidence intervals (CIs). To explore whether the bidirectional association persisted in different populations, we implemented subgroup analysis for age (45–64 and 65 years) and sex (male and female). In addition, we examined the effect modification of baseline chronic condition/sleep duration for sleep-duration analysis. Statistical analysis was performed using SPSS software (version 26), with a two-tailed *P* < 0.05 considered to be statistically significant.

## Results

### Baseline characteristics

Stage 1: Association between baseline sleep quality and follow-up multimorbidity.

The baseline characteristics of the non-multimorbid subjects are summarised in Table 1. A total of 4683 subjects were enrolled, comprising 2847 (60.79%) with good sleep quality and 1836 (39.21%) with poor sleep quality. Male sex, higher educational level, married, overweight, and current or former smoker or drinker were more likely to have experienced good sleep quality. Our data revealed that 659 (23.15%) people with good sleep quality and 566 (30.83%) with poor sleep quality developed multimorbidity during follow-up. Compare to individuals with good sleep quality, people with poor sleep quality also manifested a significantly higher prevalence of multimorbidity in the future (*P* < 0.001).

**Table 1:** Characteristics of participants at baseline in stages 1 and 2

Characteristic	Sleep quality (*N* = 4683)		*P*	Number of chronic physical conditions (*N* = 5126)		*P*
	Good (*n* = 2847)	Poor (*n* = 1836)		0 or 1 (*n* = 2847)	≥ 2 (*n* = 2279)	
Age, year			0.450			< 0.0001
45–64	2115 (74.29%)	1382 (75.27%)		2115 (74.29%)	1435 (62.97%)	
≥ 65	732 (25.71%)	454 (24.73%)		732 (25.71%)	844 (37.03%)	
Sex			< 0.0001			0.422
Male	1586 (55.71%)	749 (40.80%)		1586 (55.71%)	1244 (54.59%)	
Female	1261 (44.29%)	1087 (59.20%)		1261 (44.29%)	1035 (45.41%)	
Educational level			< 0.0001			0.003
No completion of primary school	1054 (37.02%)	819 (44.61%)		1054 (37.02%)	928 (40.72%)	
Sishu/home school/elementary school	667 (23.43%)	391 (21.30%)		667 (23.43%)	564 (24.75%)	
Middle school	730 (25.64%)	438 (23.86%)		730 (25.64%)	504 (22.11%)	
High school and above	396 (13.91%)	188 (10.24%)		396 (13.91%)	283 (12.42%)	
Residential regions			0.135			0.060
Rural	1008 (35.41%)	611 (33.28%)		1008 (35.41%)	865 (37.96%)	
Urban	1839 (64.59%)	1225 (66.72%)		1839 (64.59%)	1414 (62.04%)	
Marital status			0.002			0.026
Married	2589 (90.94%)	1618 (88.13%)		2589 (90.94%)	2030 (89.07%)	
Other	258 (9.06%)	218 (11.87%)		258 (9.06%)	249 (10.93%)	
Type of residential address			0.341			0.484
Family housing or another type	2845 (99.93%)	1833 (99.84%)		2845 (99.93%)	2276 (99.87%)	
Nursing home or hospital	2 (0.07%)	3 (0.16%)		2 (0.07%)	3 (0.13%)	
Body mass index			0.037			< 0.0001
Underweight	131 (4.60%)	93 (5.07%)		131 (4.60%)	112 (4.91%)	
Normal	1134 (39.83%)	799 (43.52%)		1134 (39.83%)	709 (31.11%)	
Overweight	634 (22.27%)	395 (21.51%)		634 (22.27%)	476 (20.89%)	
Obese	948 (33.30%)	549 (29.90%)		948 (33.30%)	982 (43.09%)	
Smoking status			< 0.0001			0.467
Non-smoker	1462 (51.35%)	1125 (61.27%)		1462 (51.35%)	1147 (50.33%)	
Current or former smoker	1385 (48.65%)	711 (38.73%)		1385 (48.65%)	1132 (49.67%)	
Alcohol consumption			< 0.0001			0.576
Non-drinker	1454 (51.07%)	1954 (57.41%)		1454 (51.07%)	1146 (50.29%)	
Current or former drinker	1393 (48.93%)	782 (42.59%)		1393 (48.93%)	1133 (49.71%)	
Social interaction			0.261			0.903
Never	1419 (49.84%)	946 (51.53%)		1419 (49.84%)	1132 (49.67%)	
At least once a month	1428 (50.16%)	890 (48.47%)		1428 (50.16%)	1147 (50.33%)	
Presence of multimorbidity			< 0.0001			
No	2188 (76.85%)	1270 (69.17%)		-	-	
Yes	659 (23.15%)	566 (30.83%)		-	-	
Ultimate condition of sleep quality						< 0.0001
Good	-	-		1842 (64.70%)	1342 (58.89%)	
Poor	-	-		1005 (35.30%)	937 (41.11%)	

Stage 2: Association between baseline chronic diseases and follow-up sleep quality.

The baseline characteristics of the subjects with good sleep quality are summarised in Tables 1 and 5126 subjects were enrolled in stage 2, with 2847 (55.54%) without multimorbidity and 2279 (44.46%) with multimorbidity. Older age, lower educational level, unmarried, underweight, and obese individuals were more likely to develop multimorbidity. One thousand five (35.30%) individuals without multimorbidity and 937 (41.11%) with multimorbidity possessed poor sleep quality during follow-up, and those with multimorbidity exhibited a significantly higher risk of poor sleep quality in the future (*P* < 0.001).

The baseline characteristics of individuals with multimorbidity and disparate sleep duration are summarised in Appendix 1, which showed that those with short sleep duration had a significantly higher prevalence of multimorbidity, while those with multimorbidity were more likely to have had short sleep duration at follow-up (*P* < 0.001).

### Prospective association between baseline sleep quality and multimorbidity at follow-up

Among people without multimorbidity, those showing poor sleep quality were associated with future multimorbidity, with a crude OR of 1.48 (1.30, 1.69). The risk of multimorbidity persisted after adjusting for covariates in the adjusted models, and the adjusted OR was 1.53 (1.33, 1.75) in the fully adjusted model. Furthermore, there was an increased response association between sleep quality and the number of developed chronic diseases (0 or 1, 2, 3, and ≥ 4). In model 3, the adjusted OR (95% CI) values were 1.39 (1.19, 1.63) for developing two diseases, 1.56 (1.23, 2.03) for developing three diseases, and 2.36 (1.68, 3.33) for developing ≥ 4 diseases (Table 2).

**Table 2:** Longitudinal association between sleep quality at baseline and risk of multimorbidity (*N* = 4683)

Number of physical conditions/ multimorbidity	OR (95% CI)			
	Unadjusted	Model 1	Model 2	Model 3
2	1.36 (1.16, 1.59)	1.36 (1.17, 1.60)	1.37 (1.17, 1.60)	1.39 (1.19, 1.63)
3	1.54 (1.21, 1.96)	1.54 (1.20, 1.97)	1.54 (1.20, 1.97)	1.56 (1.23, 2.03)
≥ 4	2.17 (1.56, 3.03)	2.23 (1.59,3.13)	2.29 (1.63, 3.21)	2.36 (1.68, 3.33)
Overall (≥ 2)	1.48 (1.30, 1.69)	1.49 (1.30, 1.70)	1.50 (1.31, 1.71)	1.53 (1.33, 1.75)

Model 1 was adjusted for sex and age. Model 2 was adjusted for sex, age, education, residential region, and marital status. Model 3 was adjusted for sex, age, education, residential regions, marital status, type of residential address, body mass index, smoking status, alcohol consumption, and social participation

### Prospective association between baseline multimorbidity and follow-up sleep quality

Among people showing good sleep quality, residents with multimorbidity were associated with future poor sleep quality, with a crude OR of 1.28 (1.14, 1.43). The risk of poor sleep quality persisted after adjusting for covariates in the adjusted models, and the adjusted OR was 1.31 (1.17, 1.47) in the fully adjusted model. Furthermore, the risk of developing poor sleep quality increased as the number of baseline chronic diseases increased. The adjusted OR (95% CI) values were 1.18 (1.02, 1.37) for two diseases, 1.27 (1.09, 1.49) for three diseases, and 2.31 (1.77, 3.00) for ≥ 4 diseases at baseline in the fully adjusted model (Table 3).

**Table 3:** Longitudinal association between multimorbidity at baseline and sleep quality (*N* = 5126)

OR (95% CI)	Number of physical conditions/multimorbidity			
	2	3	≥ 4	Overall (≥ 2)
Unadjusted	1.16 (1.00, 1.34)	1.24 (1.06, 1.44)	2.12 (1.64, 2.73)	1.28 (1.14, 1.43)
Model 1	1.18 (1.02, 1.36)	1.27 (1.09, 1.48)	2.23 (1.72, 2.89)	1.31 (1.17, 1.47)
Model 2	1.17 (1.01, 1.36)	1.26 (1.08, 1.48)	2.27 (1.75, 2.94)	1.30 (1.16, 1.46)
Model 3	1.18 (1.02, 1,37)	1.27 (1.09, 1.49)	2.31 (1.77, 3.00)	1.31 (1.17, 1.47)

Model 1 was adjusted for sex and age. Model 2 was adjusted for sex, age, education, residential region, and marital status. Model 3 was adjusted for sex, age, education, residential regions, marital status, type of residential address, body mass index, smoking status, alcohol consumption, and social interaction

### Prospective association between baseline sleep duration and follow-up multimorbidity

The relationships between night-time sleep duration and multimorbidity are presented in Table 4. A sleep duration of 6–9 h/night was set as the reference group. It was revealed that compared with the 6–9 h sleep duration, short sleep duration led to multimorbidity, with the effects persisting after adjusting the covariates that included baseline chronic condition status (OR = 1.28; 95 CI%, 1.15–1.43). However, we noted no significant difference in the future prevalence of multimorbidity between 6 and 9 h and > 9 h sleep duration.

**Table 4:** Longitudinal association between sleep duration and multimorbidity (*N* = 9823)

Sleep duration	Unadjusted		Model 1		Model 2		Model 3	
	OR (95% CI)	P	OR (95% CI)	P	OR (95% CI)	P	OR (95% CI)	P
< 6 h	1.52 (1.40, 1.65)	< 0.001	1.47 (1.35, 1.60)	< 0.001	1.47 (1.35, 1.60)	< 0.001	1.28 (1.15, 1.43)	< 0.001
6–9 h	Ref	-	Ref	-	Ref	-	Ref	-
> 9 h	1.00 (0.83, 1.21)	0.985	0.96 (0.79, 1.16)	0.670	0.97 (0.80, 1.17)	0.740	0.88 (0.67, 1.14)	0.350

Model 1 was adjusted for sex and age. Model 2 was adjusted for sex, age, education, residential regions, and marital status. Model 3 was adjusted for sex, age, education, residential regions, marital status, type of residential address, body mass index, smoking status, alcohol consumption, social participation and baseline chronic condition status

### Prospective association between baseline multimorbidity and sleep duration at follow-up

The effects of multimorbidity on night-time sleep duration are presented in Table 5. Non-multimorbidity in men and women was set as the reference group. There was a significantly higher incidence rate for individuals with multimorbidity to develop short sleep duration (< 6 h) in the future. After adjusting the covariates, this influence still remained (OR = 1.31; 95% CI, 1.20–1.42). However, multimorbidity did not exert an effect on the prolongation of sleep time.

**Table 5:** Longitudinal association between multimorbidity and sleep duration (*N* = 9823)

	Sleep duration	Unadjusted		Model 1		Model 2		Model 3	
		OR (95% CI)	*P*	OR (95% CI)	*P*	OR (95% CI)	*P*	OR (95% CI)	*P*
Multi-morbidity	< 6 h	1.41 (1,30, 1.53)	< 0.001	1.37 (1.26, 1.49)	< 0.001	1.37 (1.26, 1.48)	< 0.001	1.31 (1.20, 1.42)	< 0.001
	6–9 h	Ref	-	Ref	-	Ref	-	Ref	-
	> 9 h	1.18 (0.98, 1.44)	0.088	1.08 (0.89, 1.31)	0.447	1.08 (0.89, 1.32)	0.421	1.10 (0.89, 1.34)	0.379

Model 1 was adjusted for sex and age. Model 2 was adjusted for sex, age, education, residential regions, and marital status. Model 3 was adjusted for sex, age, education, residential regions, marital status, type of residential address, body mass index, smoking status, alcohol consumption, social participation and baseline sleep duration status

### Sensitivity analyses

Likewise, the sensitivity analyses also showed that there was a bidirectional association between sleep quality/duration and multimorbidity. The results from independent analyses by baseline disease status showed that sleep quality was associated with an increased risk of multimorbidity (Appendix 2), especially developing ≥ 4 diseases among participants with no disease (adjusted OR:7.02, 95% CI: 2.95–16.71) and those with one disease at baseline (adjusted OR:1.79, 95% CI:1.22–2.64). In addition, the results from independent analyses by baseline disease status showed that compared with the 6–9 h sleep duration, short sleep duration led to multimorbidity among participants with no disease or one disease. However, there was no significant difference in the future prevalence of multimorbidity between 6 and 9 h and > 9 h sleep durations, consistent with the results above. Moreover, the bidirectional association was robust in different demographic characteristic subgroups. The results of subgroup analysis as stratified by age, sex, and baseline chronic condition/sleep duration are shown in Appendices 4 and 5. The data revealed that the bidirectional association between sleep quality and multimorbidity was not influenced across the subgroups by either age or sex. However, the bidirectional association between sleep duration and multimorbidity still persisted except for men and those individuals with a baseline sleep duration of > 9 h.

## Discussion

In this longitudinal study in which we enrolled 9823 residents among middle-aged and older individuals from a nationally representative cohort in China, we found that there was a bidirectional association between sleep quality and multimorbidity. There was a notable dose-response association between the number of chronic diseases and sleep quality, and the bidirectional association strengthened as the number of diseases increased. We also demonstrated the bidirectional association between sleep duration and multimorbidity. Short sleep duration led to multimorbidity, and multimorbidity also resulted in short sleep duration. However, there was no significant association between long sleep duration and multimorbidity, and these findings were generally consistent after adjusting the socio-demographic, health behaviour, and lifestyle variables.

To our knowledge, this is the first-ever report of a bidirectional association between sleep duration/quality and multimorbidity in a longitudinal cohort study. Previous authors had analysed a unidirectional association between sleep quality and multimorbidity, and a majority of their studies were congruent with our study. For example, a cross-sectional study on 3250 individuals aged 60 years and older in Shanxi Province of China showed that poor sleep quality was associated with the prevalence of multimorbidity (OR = 2.445; 95% CI, 2.043–2.927) [21], and a recent longitudinal study also indicated that poor sleep quality correlated with an increased risk of multimorbidity (RR: 1.750; 95% CI, 1.476–2.076) [18]. Regarding sleep duration and multimorbidity, previous studies have shown that short sleep duration increased the risk of cardiovascular diseases [22], hypertension [23], and asthma [24]. A meta-analysis also revealed that short sleep duration was associated with numerous chronic diseases [25]. Consistent findings concerning multimorbidity were observed in both cross-sectional [26] and longitudinal studies [18]. However, there were some markedly different results in other previous studies that indicated that long sleep duration was associated with multimorbidity [27, 28]. Possible reasons for this apparent discrepancy included the authors’ different definitions of multimorbidity and the varied grouping methods adopted for sleep duration. In addition, these studies were all cross-sectional in design, such that the results may have reflected biases. Some previous studies also indicated that sleep condition was negatively impacted by diseases such as diabetes mellitus type 2 [29] and chronic kidney disease [30], illustrating an association between poor sleep condition and chronic health conditions.

Several potential reasons may also explain the bidirectional association between multimorbidity and sleep quality or duration. With respect to sleep quality, poor sleep quality leads to alterations in autonomic activity, appetite regulation, and inflammation, all of which are associated with chronic diseases [31]. Multimorbidity also increases the probability of sleep disorders [32], with the causes of poor sleep quality principally related to the chronic diseases themselves. Diabetes can lead to obstructive sleep apnoea, nocturia, and other symptoms, which in turn can lead to poor sleep quality [33]. Poor sleep quality in hypertensive patients was shown to be associated with factors such as high diastolic blood pressure and elevated blood pressure at night [34, 35]. Osteoarthritis patients may also suffer from poor sleep quality due to reduced physical activity, pain, depression, and other factors [36]. Regarding sleep duration, short sleep duration has been associated with augmented levels of the inflammatory markers C-reactive protein and interleukin-6 [37], which are also related to the onset of chronic diseases [38–40]. These authors, however, observed no relationship between the inflammatory markers and the increase in sleep duration; therefore, long sleep duration was not associated with the risk of chronic conditions. Short sleep duration may additionally influence appetite, physical activity, and thermoregulation, which in turn might lead to overweight and obesity [41]. It is conventionally acknowledged that obesity leads to an elevated risk of multimorbidity, and individuals with short sleep duration are more likely to show a higher total energy and fat intake, contributing to the development of related chronic diseases [42]. In contrast, multimorbidity raises the risk of short sleep duration—possibly due to the pain inherent to chronic diseases [43]—thus diminishing overall quality of life. Furthermore, multimorbidity is associated with stress, anxiety, and depression [44], and leads to a reduction in sleep duration. Individuals with multimorbidity are required to take numerous medications [45], and this may also exert a negative influence on their duration of sleep.

With the increasing ageing of the population, the number of patients with chronic co-morbidities is increasing and has become a serious public health problem. In order to reduce the incidence of multimorbidity, it is paramount to investigated modifiable risk factors. According to our study, sleep promotion may be a viable target for multimorbidity prevention and management [46]. Poor sleep quality or short sleep duration are modifiable risk factors for multimorbidity. Sleep disorders should be actively searched for and treated in older patients to reduce the incidence of multimorbidity. Currently, the problems of sleep disorders in older patients in China remain prominent. A study on the sleep quality of older patients with different chronic diseases in China indicated that only 9.9% of patients manifested favourable sleep quality [47]. A large-scale multi-centre survey also revealed that the overall prevalence of insomnia in the older people over 65 years of age in China was 38.54% [48]. However, as there are few extant studies on sleep management in older patients, current research on sleep quality management in the older population in China needs to be bolstered [49, 50]. So more attention should be given to sleep disorders improvement, in order to prevent or reduce a number of chronic diseases.

In this study, we validated the bidirectional association between sleep quality or duration and multimorbidity in middle-aged and older Chinese adults through a longitudinal study. However, there were some limitations to this study. First, the information provided on multimorbidity was entirely self-reported, potentially leading to measurement error. However, based on a previous study, self-reported multimorbidity shows good specificity in large population-based studies [51]. Second, there was only one question on sleep quality, which might possibly contribute to a bias due to inaccurate subjective judgement. Nevertheless, there was no further information available in the CHARLS, and future studies should therefore encompass measurements of sleep quality from multiple perspectives. Moreover, because most of the missing data were missing individual chronic conditions and sleep condition information, which were the main elements analysed in this paper, so there was no way to carry out sensitivity analyses to account for missing data, the paper does not provide a full picture of the importance of sleep for multimorbidity. In the future, an in-depth analysis of the issue could be attempted using more comprehensive database.

## Conclusion

We herein showed for the first time that there exists a bidirectional association between sleep quality or short sleep duration and multimorbidity in middle-aged and older Chinese adults. Based on this finding, promoting sleep may be an effective way to prevent and manage multiple diseases, so we recommend that additional attention be given to sleep condition among middle-aged and older people. Healthcare institutions, especially primary care providers, should actively seek out and treat sleep disorders in elderly patients to reduce the incidence of multimorbidity. In addition, patients with multimorbidity usually have a poor sleep condition, so we also posit that primary care should enhance awareness of sleep condition in patients with multimorbidity to improve their quality of life. More attention should be paid on the study of sleep management, not only prevents and reduces the incidence of multimorbidity, but also improves the quality of life of patients with multimorbidity.

### Electronic supplementary material

Below is the link to the electronic supplementary material.

Supplementary Material 1

## Data Availability

The data of this study are available at [http://charls.pku.edu.cn/index/en.html]. [The CHARLS] Yaohui Zhao, et al.; 2018; Harmonized CHARLS; the Gateway to Global Aging Data; Version C; http://charls.pku.edu.cn/pages/data/harmonized_charls/en.html.
