# Supplementary material for: Bidirectional associations between sleep quality/duration and multimorbidity in middle-aged and older people Chinese adults: a longitudinal study
Source: BMC Public Health. 2024 Mar 5;24:708. doi: 10.1186/s12889-024-17954-8 (PMC10916205; doi:10.1186/s12889-024-17954-8)
Supplement: Supplementary file 1 — Supplementary Material 1 [file 12889_2024_17954_MOESM1_ESM.docx]

Appendix 1 Characteristics of participants for sleep duration and chronic physical conditions at baseline.

| Characteristic | Sleep duration in 2015(N =9823) | | | *P* | Number of chronic physical  conditions in 2015 (N =9823) | | *P* |
| --- | --- | --- | --- | --- | --- | --- | --- |
|  | <6h  (n=2847) | 6-8h  (n=1836) | >8h  (n=1836) |  | 0 or 1(n=2847) | ≥ 2(n=2279) |  |
| Age, year |  |  |  | <0.0001 |  |  | <0.0001 |
| 45-64 | 3319(66.78%) | 3143(71.69%) | 303(64.61%) |  | 3497(74.67%) | 3268(63.58%) |  |
| ≥65 | 1651(33.22%) | 1241(28.31%) | 166(35.39%) |  | 1186(25.33%) | 1860(36.42%) |  |
| sex |  |  |  | <0.0001 |  |  | <0.0001 |
| Male | 2179(43.84%) | 2214(50.50%) | 216(46.06%) |  | 2335(49.86%) | 2274(44.24%) |  |
| Female | 2719(56.16%) | 2170(49.50%) | 253(53.94%) |  | 2348(50.14%) | 2866(55.76%) |  |
| Education level |  |  |  | <0.0001 |  |  | <0.0001 |
| Did not finish primary school | 2234(44.95%) | 1729(39.44%) | 231(49.25%) |  | 1873(40.00%) | 2321(45.16%) |  |
| Sishu/home school/elementary school | 1147(23.08%) | 1040(23.72%) | 116(24.73%) |  | 2018(22.59%) | 1245(24.22%) |  |
| Middle school | 1064(21.41%) | 1049(23.93%) | 85(18.12%) |  | 1168(24.94%) | 1030(20.04%) |  |
| High school and above | 525(10.56%) | 566(12.91%) | 37(7.89%) |  | 584(12.47%) | 544(10.58%) |  |
| Residential regions |  |  |  | <0.0001 |  |  | 0.294 |
| Rural | 1754(35.29%) | 1528(36.09%) | 112(23.88%) |  | 1619(34.57%) | 1829(35.58%) |  |
| Urban | 3216(64.71%) | 2802(63.91%) | 357(76.12%) |  | 3064(65.43%) | 3311(64.42%) |  |
| Marital status |  |  |  | <0.0001 |  |  | <0.0001 |
| Married | 4303(86.58%) | 3956(90.24%) | 402(85.71%) |  | 4207(89.84%) | 4454(86.65%) |  |
| Other | 667(13.42%) | 428(9.76%) | 67(14.29%) |  | 476(10.16%) | 686(13.35%) |  |
| Type of Residential Address |  |  |  | 0.497 |  |  | 0.188 |
| Family housing or another type | 4960(99.80%) | 4378(99.86%) | 469(100%) |  | 4678(99.89%) | 5129(99.79%) |  |
| Nursing home or hospital | 10(0.20%) | 6(0.14%) | 0(0%) |  | 5(0.11%) | 11(0.21%) |  |
| Body mass index |  |  |  | 0.005 |  |  | <0.0001 |
| Underweight | 286(5.75%) | 194(4.43%) | 32(6.82%) |  | 224(4.78%) | 288(5.60%) |  |
| Normal | 1871(37.65%) | 1592(36.31%) | 183(39.02%) |  | 1933(41.28%) | 1713(33.33%) |  |
| Overweight | 1068(21.49%) | 934(21.30%) | 97(20.68%) |  | 1029(21.97%) | 1070(20.82%) |  |
| Obese | 1745(35.11%) | 1664(37.96%) | 157(33.48%) |  | 1497(31.97%) | 2069(40.25%) |  |
| Smoking status |  |  |  | 0.143 |  |  | 0.025 |
| Nonsmoker | 2851(57.36%) | 2426(55.34%) | 265(56.50%) |  | 2587(55.24%) | 2955(57.49%) |  |
| Current or former smoker | 2119(42.64%) | 1958(44.66%) | 204(43.50%) |  | 2096(44.76%) | 2185(42.51%) |  |
| Alcohol consumption |  |  |  | 0.228 |  |  | 0.303 |
| Nondrinker | 2721(54.75%) | 2331(53.17%) | 262(55.86%) |  | 2508(53.56%) | 2806(54.59%) |  |
| Current or former drinker | 2249(45.25%) | 2053(46.83%) | 207(44.14%) |  | 2175(46.44%) | 2334(45.41%) |  |
| Social participation |  |  |  | 0.011 |  |  | 0.592 |
| Never | 2532(50.95%) | 2142(48.86%) | 259(55.22%) |  | 2365(50.50%) | 2568(49.96%) |  |
| At least once a month | 2438(49.05%) | 2242(51.14%) | 210(44.78%) |  | 2318(49.50%) | 2572(50.04%) |  |
| Final presence of multimorbidity |  |  |  | <0.0001 |  |  |  |
| No | 1790(36.02%) | 2021(46.10%) | 216(46.06%) |  | - | - |  |
| Yes | 3180(63.98%) | 2363(53.90%) | 253(53.94%) |  | - | - |  |
| Final presence of sleep duration |  |  |  |  |  |  | <0.0001 |
| <6h | - | - |  |  | 2395(51.14%) | 3039(59.12%) |  |
| 6-8h | - | - |  |  | 2066(44.12%) | 1864(36.27%) |  |
| >8h |  |  |  |  | 222(4.74%) | 237(4.61%) |  |

Appendix 2 Longitudinal association between sleep quality at baseline and risk of single disease and multimorbidity by baseline status

| Number of physical conditions/ multimorbidity | OR (95% CI) | | | |
| --- | --- | --- | --- | --- |
|  | Unadjusted | Model 1 | Model 2 | Model 3 |
| No disease at baseline | | | | |
| 1 | 1.18 (0.96, 1.46) | 1.21 (0.98, 1.50) | 1.21 (0.98, 1.50) | 1.22 (0.99, 1.51) |
| 2 | 1.57 (1.13, 2.18) | 1.59 (1.14, 2.22) | 1.60 (1.14, 2.24) | 1.64 (1.17, 2.29) |
| 3 | 1.36 (0.79, 2.33) | 1.36 (0.78, 2.38) | 1.38 (0.79, 2.41) | 1.36 (0.78, 2.39) |
| ≥4 | 4.76 (2.07, 10.97) | 6.09 (2.62, 14.18) | 6.26 (2.67, 14.66) | 7.02 (2.95, 16.71) |
| One disease at baseline | | | | |
| 1 | - | - | - | - |
| 2 | 1.23 (1.02, 1.48) | 1.23 (1.02, 1.49) | 1.25 (1.03, 1.50) | 1.27 (1.05, 1.53) |
| 3 | 1.48 (1.12, 1.96) | 1.49 (1.12, 1.97) | 1.48 (1.11, 1.97) | 1.56 (1.17, 2.07) |
| ≥4 | 1.71 (1.18, 2.49) | 1.67 (1.14, 2.43) | 1.74 (1.19, 2.54) | 1.79 (1.22, 2.64) |

Model 1 was adjusted for sex and age. Model 2 was adjusted for sex, age, education, residential region, and marital status. Model 3 was adjusted for sex, age, education, residential regions, marital status, type of residential address, body mass index, smoking status, alcohol consumption, and social participation.

Appendix 3 Longitudinal association between sleep at baseline and risk of single disease and multimorbidity by baseline status

| Number of physical conditions/ multimorbidity | | OR (95% CI) | | | |
| --- | --- | --- | --- | --- | --- |
|  |  | 1 | 2 | 3 | ≥4 |
| No disease at baseline | | | | | |
| <6 h | Unadjusted | 1.02 (0.84, 1.26) | 1.52 (1.09, 2.13) | 1.70 (0.98, 2.94) | 2.83 (1.22, 6.56) |
|  | Model 1 | 1.00 (0.82, 1.24) | 1.49 (1.07, 2.09) | 1.58 (0.91, 2.75) | 2.80 (1.21, 6.51) |
|  | Model 2 | 0.99 (0.81, 1.22) | 1.45 (1.03, 2.03) | 1.55 (0.89, 2.70) | 2.73 (1.17, 6.35) |
|  | Model 3 | 1.00 (0.81, 1.22) | 1.45 (1.04, 2.04) | 1.55 (0.89, 2.72) | 2.79 (1.19, 6.53) |
| 6–9 h |  | - | - | - | - |
| >9 h | Unadjusted | 0.90 (0.56, 1.47) | 1.05 (0.46, 2.39) | 1.39 (0.41, 4.77) | 1.33 (0.16, 10.85) |
|  | Model 1 | 0.89 (0.54, 1.44) | 1.06 (0.46, 2.41) | 1.17 (0.34, 4.09) | 1.45 (0.18, 11.92) |
|  | Model 2 | 0.88 (0.54, 1.43) | 1.03 (0.45, 2.36) | 1.14 (0.32, 4.12) | 1.43 (0.17, 11.96) |
|  | Model 3 | 0.89 (0.54, 1.46) | 1.07 (0.47, 2.46) | 1.32 (0.36, 4.82) | 1.61 (0.18, 14.63) |
| One disease at baseline | | | | | |
| <6 h | Unadjusted | - | 1.03 (0.86, 1.25) | 1.30 (0.97, 1.73) | 1.70 (1.15, 2.51) |
|  | Model 1 | - | 1.03 (0.86, 1.25) | 1.29 (0.96, 1.72) | 1.65 (1.12, 2.44) |
|  | Model 2 | - | 1.04 (0.86, 1.25) | 1.28 (0.96, 1.71) | 1.67 (1.13, 2.47) |
|  | Model 3 | - | 1.03 (0.85, 1.25) | 1.32 (0.98, 1.76) | 1.67 (1.12, 2.47) |
| 6–9 h |  | - | - | - | - |
| >9 h | Unadjusted | - | 0.81 (0.52, 1.26) | 1.20 (0.65, 2.23) | 1.00 (0.39, 2.58) |
|  | Model 1 | - | 0.81 (0.52, 1.26) | 1.18 (0.63, 2.20) | 0.95 (0.37, 2.46) |
|  | Model 2 | - | 0.81 (0.52, 1.27) | 1.24 (0.66, 2.32) | 0.96 (0.37, 2.50) |
|  | Model 3 | - | 0.79 (0.50, 1.24) | 1.23 (0.65, 2.31) | 0.91 (0.35, 2.39) |

Model 1 was adjusted for sex and age. Model 2 was adjusted for sex, age, education, residential region, and marital status. Model 3 was adjusted for sex, age, education, residential regions, marital status, type of residential address, body mass index, smoking status, alcohol consumption, and social participation.

Appendix 4 Subgroups analyses on the associations of sleep quality and duration with the risk of multimorbidity.

|  | Age^a^ | | sex^b^ | | Baseline chronic condition^c^ | |
| --- | --- | --- | --- | --- | --- | --- |
|  | 45-64 | ≥65 | Male | Female | 0 or 1 | ≥2 |
| Sleep quality |  |  |  |  |  |  |
| Good | Ref | - | Ref | - | Ref | - |
| Poor | 1.50(1.28,1.76) | 1.64(1.28,2.12) | 1.46(1.20,1.77) | 1.58(1.31,1.90) | - | - |
| Sleep duration |  |  |  |  |  |  |
| <6h | 1.32(1.16,1.50) | 1.39(1.14,1.70) | 1.09(0.93,1.27) | 1.56(1.34,1.83) | 1.30(1.13,1.48) | 1.44(1.20,1.72) |
| 6-8h | Ref | - | Ref | - | Ref | - |
| >8h | 0.80(0.58,1.09) | 1.15(0.75,1.75) | 1.04(0.72,1.50) | 0.85(0.60,1.21) | 0.99(0.72,1.36) | 0.80(0.55,1.17) |

^a^ Adjusted for sex, education, residential regions, marital status, type of residential address, body mass index, smoking status, alcohol consumption, social participation(and baseline chronic condition).

^b^ Adjusted for age, education, residential regions, marital status, type of residential address, body mass index, smoking status, alcohol consumption, social participation (and baseline chronic condition).

^c^ Adjusted for sex, age, education, residential regions, marital status, type of residential address, body mass index, smoking status, alcohol consumption, social participation.

Appendix 5 Subgroups analyses on the associations of multimorbidity with sleep quality and duration.

| Multi-  morbidity |  | Age^a^ | | sex^b^ | | Baseline sleep duration^c^ | | |
| --- | --- | --- | --- | --- | --- | --- | --- | --- |
|  |  | 45-64 | ≥65 | Male | Female | <6h | 6-8h | >8h |
|  | Sleep quality |  |  |  |  |  |  |  |
|  | Good | Ref | - | Ref | - | Ref | - | - |
|  | Poor | 1.29  (1.12,1.48) | 1.35  (1.09,1.68) | 1.32  (1.13,1.56) | 1.30  (1.10,1.54) | - | - | - |
|  | Sleep duration |  |  |  |  |  |  |  |
|  | <6h | 1.30  (1.16,1.44) | 1.21  (1.02,1.43) | 1.34  (1.17,1.52) | 1.21  (1.07,1.37) | 1.18  (1.04,1.34) | 1.40  (1.22,1.60) | 1.07  (0.68,1.70) |
|  | 6-8h | Ref | - | Ref | - | Ref | - | - |
|  | >8h | 1.14  (0.88,1.49) | 1.04  (0.76,1.41) | 0.9  (0.73,1.30) | 1.19  (0.90,1.59) | 1.14  (0.87,1.50) | 1.17  (0.79,1.73) | 0.91  (0.55,1.49) |

^a^ Adjusted for sex, education, residential regions, marital status, type of residential address, body mass index, smoking status, alcohol consumption, social participation(and baseline sleep duration).

^b^ Adjusted for age, education, residential regions, marital status, type of residential address, body mass index, smoking status, alcohol consumption, social participation (and baseline sleep duration).

^c^ Adjusted for sex, age, education, residential regions, marital status, type of residential address, body mass index, smoking status, alcohol consumption, social participation.
